# Flow and Physiological Response Assessment during Exercise Using Metrorhythmic Stimuli

**DOI:** 10.5114/jhk/187804

**Published:** 2024-07-17

**Authors:** Damian Kania, Patrycja Romaniszyn-Kania, Monika Bugdol, Aleksandra Tuszy, Daniel Ledwoń, Anita Pollak, Andrzej W. Mitas

**Affiliations:** 1 Institute of Physiotherapy and Health Sciences, The Jerzy Kukuczka Academy of Physical Education in Katowice, Katowice, Poland.; 2 Faculty of Biomedical Engineering, Silesian University of Technology, Zabrze, Poland.; 3 Institute of Psychology, University of Silesia in Katowice, Katowice, Poland.

**Keywords:** rhythmic auditory stimulation, physiological response, flow assessment, physical activity, physiological signals

## Abstract

Activity and physical effort positively affect a person's psychophysical state and emotional experience. Interest in the phenomenon of flow, the state of perceived arousal, stems from its relationship to an individual's intrinsic motivation. Flow is an area of research in many fields, including sports. Nowadays, solutions are being sought to support the traditional assessment of cognitive and affective states using analysis of physiological signals. Therefore, the present study analysed and estimated the physiological responses that may occur during the induction of a flow state between exercises stimulated by additional metrorhythmic stimuli. Thirty-six healthy subjects participated in the study. The effects of various metrorhythmic stimuli on the body's physiological response during the subjects' free gait were examined. The physiological response and flow intensity were evaluated when the rate of individual stimuli was changed, and the rate was enforced. Several statistically significant variables and correlations were determined for physiological indicators depending on the stage of the study conducted and the level of flow experienced. A positive, statistically significant correlation of flow and HRV frequency variables was obtained. The results also confirm previous literature reports on the relationship between flow response and frequency heart rate variability during physical activity.

## Introduction

The available literature confirms the positive impact of physical activity on a person's psychophysical state and emotional experience (An et al., 2020). According to the intensity-affect-adherence chain, people participate in those physical activities they enjoy, and lower-intensity exertion is associated with higher levels of pleasure (Oliveira et al., 2015). However, the rate of performing a given physical activity does not always match an individual's preferences. Negative effects can result from persuading individuals to increase or decrease. It has been shown that if the level of intensity is increased or decreased by 10% relative to that chosen by the exerciser, this leads to a decrease in the feeling of pleasure (Lind et al., 2008).

A primary role in promoting positive and effective forms of motivation in physical exercise can be provided by metrorhythmic stimulations of a precisely defined character, tempo, and melodiousness (Jebabli et al., 2022; Zenter et al., 2008). Rhythm, a fundamental structural feature of music, involves sensory perception and motor entrainment, resulting in complex cognitive functions and motor adaptations (Benitez-Burraco and Murphy, 2016). In addition, rhythmic entrainment affects the heart rate, contributes to pain reduction and muscle relaxation (Bengtsson et al., 2009). On the other hand, the affective-aesthetic response to a given stimulus includes arousal, motivation and production of emotions (Thaut et al., 2009). Inherently related to the individual's intrinsic motivation is the phenomenon of flow (Csikszentmihalyi, 1997). It is a psychological state in which a person simultaneously feels cognitively effective, motivated, and happy, with a neurophysiological background (Csikszentmihalyi, 1997). Consequently, flow being, according to the flow-channel model, a state between boredom and anxiety, should be associated with moderate physiological arousal due to autonomic nervous system (ANS) activation (Peifer et al., 2014). Numerous research efforts are being conducted to determine objective measures and understand the body's physiological response to experienced flow, such as by determining heart rate variability (HRV) (Irshad et al., 2023; Peifer et al., 2015; Tozman et al., 2015). According to LeDoux and Bemporad (1997), the physiological response of the body to emotional reactions assumes that an indispensable element of any emotional process is the affect, which is generated automatically and involuntarily due to the stimulation of the amygdala (LeDoux and Bemporad, 1997). The amygdala, responsible for the body's defensive reactions, including reactions to emotions, stimulates the Sudomotor Nervous System (SNS). Thus, the eccrine sweat glands, responsible for thermoregulation, are activated. All this contributes to changes in skin conductivity and the generation of higher electrodermal activity (EDA) signals (Boucsein, 2012). SNS stimuli also affect the heart rate. A relative increase in sympathetic activity causes a reduction in the time between heartbeats (the beat-to-beat interval), and a relative increase in parasympathetic activity causes lengthening of the R-R interval. This is reflected in the blood volume pulse (BVP) or heart rate variability (HR) (Benarroch, 1993).

Solutions are increasingly being developed to support the traditional assessment of cognitive and affective states using analysis of physiological signals, i.e., EDA, BVP, HRV, and EEG (González-Hernández et al., 2023; Park et al., 2019; Prończuk et al., 2023). Based on EDA signals, BVP, and Deep Learning methods, a study by Maier et al. (2019) presents a system for estimating flow levels and distinguishing affective states (boredom, stress). Other research focused on the physiological activity of users in the flow state (Tian et al., 2017). A study by Sweetser and Wyeth (2005) pointed out the importance of the flow effect in experiencing pleasure from games. Di Lascio et al. (2021) undertook research into using physiological data (from wristband devices) in conjunction with contextual information (self-report questionnaires) to automatically distinguish between low and high flow levels. A study by Jha et al. (2022) verified how autonomic-cardiac modulation was related to the flow effect during instrument playing by professional musicians. Elbe et al. (2010) examined how physical activity affected experienced flow and whether there was a relationship between flow and improved physiological response during exercise sessions. The flow level was assessed for explicit feedback using multiple modalities while playing tennis (Havlucu et al., 2018). Psychological correlates of flow as a trait and disposition in professional athletes were also examined (Jackson et al., 1998). Studies indicate that more frequent play of popular exergames and more prolonged use of game consoles can increase the frequency of flow and pleasure, directly affecting the sense of physical fitness (Lai et al., 2012).

To the best of the authors' knowledge, there has been no analysis of the flow phenomenon in the literature, based on physiological signals during the performance of physical activity, especially activity with metrorhythmic stimuli. Therefore, the present research aimed to analyse the body's physiological response to various types of metrorhytmic stimuli given during physical activity and to search for the relationship between the determined physiological response of the body and the induction of the flow state.

## Methods

### Participants

The study group comprised 36 healthy participants (15 women, 21 men) aged between 20 and 38 years (22.03 ± 3.40). After informing participants about the nature of the conducted experiment, each participant provided voluntary written informed consent. Individuals with musculoskeletal dysfunction, those exhibiting a pathological gait, those with a history of hearing disorders, or any neurological or psychiatric disorders during their lifetime were excluded from the experiment. The ethics committee of the Jerzy Kukuczka Academy of Physical Education in Katowice approved the study protocol (approval code: 01/2023; approval date: 29 June 2023), which was in accordance with the Declaration of Helsinki.

### Study Design and Data Acquisitions

**Figure 1:**
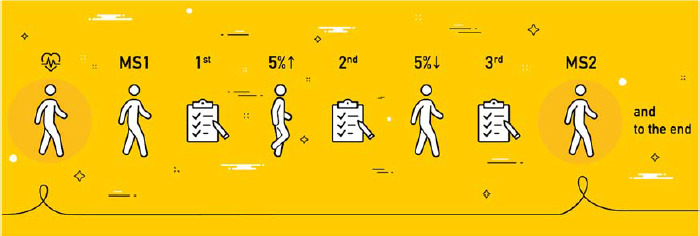
Schematic of the measurement protocol.

The research protocol was divided into stages. A schematic view of the conducted research is shown in Figure 1.

The experimental stage consisting of examining responses to specific stimuli took place in the Department of Biomedical Engineering laboratory at the Silesian University of Technology. First, the effect of different types of metrorhythmic stimuli on physiological variables during the free gait of participants was tested (first stage of the subsequent analysis: different types of stimulation). Next, it was examined how the change in the rate of individual stimuli and the imposed rate of the gait affected the physiological responses (second stage of analysis: a different tempo) and the severity of flow in participants (third stage: flow and the physiological response).

Before the experiment, the participant was provided with the measurement equipment, i.e., an Empatica E4 device recording physiological signals such as electrodermal activity (EDA), body temperature, the accelerometric signal from three axes (ACC X/Y/Z) and the blood volume pulse (BVP), from which heart rate variability (HRV) and heart rate (HR) variables were determined at the processing stage (Empatica, 2015). Then, the participant was asked to walk freely around the lab for about 2 min.

In the next step of the research protocol, based on the free gait tempo determined in the previous step, the participant's task was to attempt to synchronise his/her gait with the metrorhythmic stimuli set—MS1 (metronome sound no. 1). For this purpose, the proband was put on headphones (Beyerdynamic DT770 Pro 80Ohm) connected to a smartphone, on which the metronome app was running. On the instruction of the researcher, the participant began a free gait (6 laps along a lab-designated path) with an attempt to synchronise to a set sound with a meter of ¼ in a tempo determined from the first trial.

Afterwards, the participant was asked to fill out a flow questionnaire about experiences during this study stage. The Flow Frequency Scale questionnaire consisting of 11 items allows to evaluate on a scale of 1 (never) to 6 (always), how often flow was experienced during a given exercise (Bartzik et al., 2021). This protocol element was repeated each time the experiment was performed with a different configuration of metrorhythmic stimuli.

Due to the change in the participant's type of stimulation, the tempo of the inflicted metrorhythmic beat was increased by 5% relative to the initial value (metronome sound no. 1 105%. MS1_105_). Subsequently, the tempo was decreased by 5% in the next trial (metronome sound no. 1 95%. MS1_95_), according to the approach in MS1. After completion of the trials, participants were also asked to fill out the flow questionnaire. In the second approach, the steps were repeated, however, the presented stimulus changed. The approach used a different, less monotonous sound in 4/4 meter, with a distinct accent on every 4^th^ beat of the metronome (MS2, MS2_105_, and MS2_95_).

After completing the surveys, the participant was removed from the headphones and the Empatica E4 device.

### Data Analysis

#### Socio-Economical Status and Psychological Analysis

Csikszentmihalyi's characteristics (2000), as reflected in the Flow Frequency Scale, indicate a frequency of experiencing flow, a state of pleasure and total absorption while performing activities. Clear feedback, clear goals, and balance between demands and capabilities facilitate flow. Absorption is associated with being focused, deeply engaged, highly motivated, confident, and acting spontaneously, even in an almost automatic way. The score represents the sum of all responses marked by the respondent. A higher score corresponds with a higher frequency of experiencing flow.

For the analysis of the questionnaire obtained in stages 1 and 2, the indications of participants were summed up and then the average was calculated. The distribution conformity analysis revealed a right-skewed distribution. The results in all samples were high (5.139 < M < 5.336), exceeding the theoretical mean and approaching the maximum score.

The reliability analysis using the Cronbach's alpha coefficient showed satisfactory reliability in each measurement (Table 1).

**Table 1:** The reliability of the flow scale during the relevant stages of the protocol based on the Cronbach's alpha coefficient (N = 29).

	M	SD	Me	Minimum	Maximum	Cronbach’s α
FlowMS1	5.139	0.645	5.409	37	65	0.80
FLOWMS1_105	5.288	0.571	5.545	45	66	0.73
FLOWMS1_95	5.162	0.689	5.455	39	66	0.86
FLOWMS2	5.336	0.596	5.591	45	66	0.78
FLOWMS2_105	5.313	0.676	5.545	36	66	0.87
FLOWMS2_95	5.177	0.754	5.409	36	66	0.88

#### Signal Preprocessing

During the experiment, the Empatica E4 device continuously recorded physiological signals. Temporal markers were introduced during the recording, dividing each signal into individual segments (experiments MS1 through MS295) which were independently analysed. Preprocessing was based on previous scientific reports on examining physiological signals during exercise to analyse a subjects's emotionality (Romaniszyn-Kania, 2020, 2021).

The first signal analysed was electrodermal activity. Determination of the basic components of EDA (tonicity, phasicity, and additive error) made it possible to determine the most relevant indicators, which are the skin conductance level (SCL) and galvanic-skin response (GSR) (Greco et al., 2015). The following variables were estimated based on the obtained values: response GSRs per minute (rpm), the total number and the average amplitude of GSRs for a given experiment stage, and the number of significant GSRs (with amplitude greater than 1.5 uS). In addition, a set of time-domain statistical features including mean, standard deviation, median, variance, percentiles 25 and 75, quartile deviation, minimum, maximum, and range of the signal, fourth and fifth order moments, skewness, kurtosis, mean squared error, entropy, the slope coefficient to the regression line, signal tone, and regression line shift coefficients, was determined for each signal section. The next step of signal preprocessing included Blood Volume Pulse (BVP) analysis. BVP signal features such as fundamental frequency f0, mean frequency fm, bandwidth (BP), and the centre of gravity (CoG) were determined. Subsequently, BVP was the basis for defining a series of time coefficients describing heart rate variability such as Standard Deviation of Successive Differences (SDSD), Standard Deviation of Normal-to-Normal Intervals (SDNN), Root Mean Square of Successive Differences (RMSSD), probability of intervals greater than 50 ms (pNN50), Triangular Interpolation of NN Interval Histogram (TINN), Heart Rate Variability Triangular Index (TRI), and mean heart rate (HR). In addition, Spectral density for very low frequency (VLF), spectral density for low frequency (LF), spectral density for high frequency (HF), and LF to HF power ratio (LF/HR ratio) were calculated in the frequency domain. Forty-six physiological features were determined at each study stage with metrorhythmic beats.

#### Statistical Analysis

Group comparisons were preceded by verifying normality (Shapiro-Wilk test) and evaluating variances equality (F test and Levene’s test for two or more groups, respectively). If possible, parametric tests were used (*t*-Student); otherwise, non-parametric alternatives were employed (U Mann-Whitney). In post-hoc analysis, the pairwise *t*-test and the pairwise Wilcoxon test for unpaired samples with Holm correction were used. For each music genre, a binary group was created (1: prefers this genre, 0: does not prefer this genre), making it possible to compare independent groups for psychological variables. The correlation was assessed based on the Pearson coefficient. The relationships between nominal variables were evaluated using the chi-squared independence test. Missing data imputation was performed using the k-NN (k-Nearest Neighbors) method and only for those variables for which less than 5% of information was missing. The level of significance was set to α = 0.05. The analysis was performed in R 4.2.1 in the RStudio environment using package stats, afex, e1071, tidyverse, rstatix, impute, car, rcompanion.

## Results

### Different Types of Stimulation

**Figure 2:**
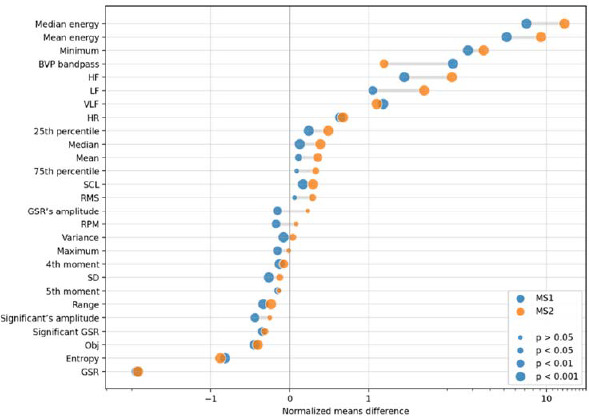
Mean values of variables showing statistically significant differences in each stage of the study; unit is a standard deviation.

The first part of the analysis involved searching for differences between the characteristics of physiological signals determined during the various stages of the experiment. It allowed to compare the results obtained during the free gait (determination of baseline for each person) and the gait with the first type of metrorhythmic arousal along with the second modified type. Figure 2 presents the mean values of variables showing statistically significant differences between baseline (gray, horizontal line), MS1 (orange circle) and MS2 (blue circle). To present statistically significant results, the obtained values of physiological variables were normalised using the z-score method.

### Different Tempos of Stimulation

**Figure 3:**
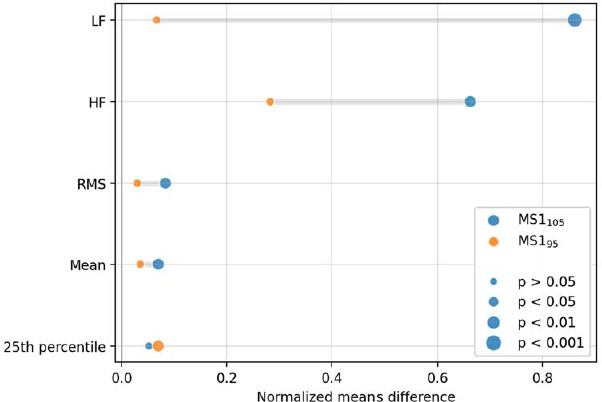
Mean values of variables showing statistically significant differences in the stages of tests using metrorhythmic stimulation no. 1.

Subsequently, it was verified how the participant's response changed depending on the type of the stimulus presented. Intrapersonal correlations were checked for each physiological trait measured at each trial stage. Statistically significant differences were determined between MS1-MS1_105_ and MS1-MS1_95_ trials for physiological variables (Figure 3).

In addition, statistically significant differences between stage MS1105 and MS195 were observed for frequency variables such as LF (*p* = 0.008), HF (*p* = 0.028), and LF/HF (*p* = 0.029). The flow level was also significantly different among the three consecutive measurements: FLOW_MS1_, FLOW_MS1_105_ and FLOW_MS1_95_ (*p* = 0.031).

**Figure 4:**
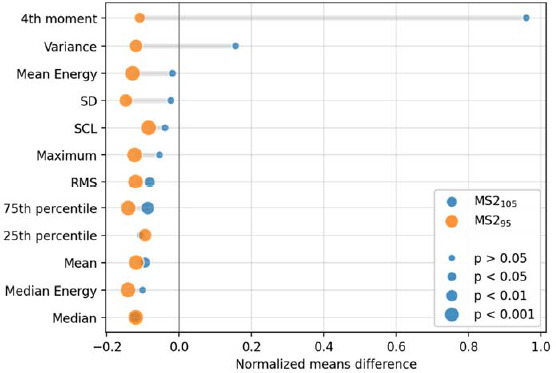
Mean values of variables showing statistically significant differences in the stages of trials using type no. 2 of metrorhythmic stimulation.

Significant differences were determined between MS2-MS2_105_ and MS2-MS2_95_ trials. The significance of variables is shown in Figure 4.

Due to comparing the values of the determined features between the MS2_105_ and MS2_95_ stages, statistically significant differences were obtained for HRV frequency variables such as LF (*p* = 0.012), HF (*p* = 0.028) and LF/HF (*p* = 0.029). No significant differences were confirmed between flow measurements.

### Flow and the Physiological Response

As part of subsequent analysis of the relationship between physiological phenomena and responses that might manifest in inducing the flow phenomenon between tasks stimulated additionally with metrorhythmic stimuli, it was verified whether, and if so, which characteristics differentiated the group of participants in terms of the level of perceived flow (below or above the median). Statistically significant differences were obtained for minimum (*p* = 0.0309) and maximum (*p* = 0.0349) values of the electrodermal activity signal as well as for kurtosis (*p* = 0.0486). Significantly higher skin conductance levels (*p* = 0.0398) were shown in participants experiencing higher flow levels. The amplitude of skin-galvanic responses was also significantly higher in this group (*p* = 0.0413).

Next, the correlation of physiological features with responses regarding the flow effect was analysed (Table 2). It was shown that flow for consecutive measurements significantly correlated mainly with heart rate variability variables such as TINN, SDNN, pNN50, HR value, and HRV frequency variables: LF, HF, VLF, LF/HF ratio. Statistically significant moderate correlations were found for FLOW_MS1_ and TINN (r = −0.42), FLOW_MS2_105_, and CoG variables (r = 0.47). In addition, correlations of the electrodermal activity signal (Obj) with FLOW_MS2_105_ (r = 0.33) were also shown.

**Table 2:** Coefficients of significant correlations of flow and physiological variables.

	HRV								EDA
Stage	**TINN**	**SDNN**	**pNN50**	**CoG**	**LF**	**HF**	**LF/HF**	**HR**	**Obj**
FLOW_MS1_	**−0.42**								
FLOW_MS1_105_					0.35	0.35	−0.35	0.33	
FLOW_MS1_95_	−0.33								
FLOW_MS2_		−0.34							
FLOW_MS2_105_			0.39	**0.47**					0.33
FLOW_MS2_95_	−0.34								

Note: HRV: heart rate variability; EDA: electrodermal activity; MS1: metronome sound no. 1; MS1_105: metronome sound no. 1 105% of the basic metrorhythmic beat; MS1_95: metronome sound no. 1 95% of the basic metrorhythmic beat; MS2: metronome sound no. 2; MS2_105: metronome sound no. 2 105% of the basic metrorhythmic beat; MS2_95: metronome sound no. 2 95% of the basic metrorhythmic beat; TINN: Triangular Interpolation of NN Interval Histogram; SDNN: Standard Deviation of Normal-to-Normal Intervals; pNN50: probability of intervals greater than 50 ms; CoG: center of gravity; LF: spectral density for low frequency; HF: spectral density for high frequency; LF/HF: LF to HF power ratio; HR: heart rate; Obj: quadratic metric of the discrepancy between predicted and observed data in the electrodermal activity signal

## Discussion

Physical activity is an important and indispensable part of everyday life. The motives for undertaking it and the accompanying emotions are the research topics of scientists worldwide. Within the scope of the present work, a study with fundamental objectives was carried out. An analysis was undertaken of the physiological effects and reactions that can manifest themselves during physical activity additionally stimulated by metrorhythmic stimuli, as well as the induction of the flow effect.

It should be noted that the relevant indicators determined from physiological signals are repeated during individual activities. Given the specificity of measuring psychological variables, data collected through declarative methods allow for capturing an individual's interpretation of his/her reaction rather than the reaction that could be generalized within a wider context (Poels and Dewitte, 2006). Most of the differentiating features, comparing baseline vs. MS1 and MS2, were obtained from the electrodermal activity signal (Obj, SLC, mean value, median, SD, 25^th^ and 75^th^ percentile, variance, minimum and maxim value of EDA, range). These variables were successively higher in subsequent stages. This was related to the participant’s physical activity and the increasingly higher level of activation of the SNS system by metrorhythmic stimulation. Other studies have also identified the basic vriables of the EDA signal as differential when attempting to synchronise the gait to the presented auditory stimulus (Mańka et al., 2021). The differences between baseline vs. MS1 and baseline vs. MS2 included also differential HRV frequency ratios (VLF, LF, HF), possibly due to the severity of flow during a given task. Apart from the issue of physical activity, it has also been proven in the literature that, compared to silence, auditory stimuli (including simple metronome tones) may induce increases and decreases in HRV variables. The main element causing the effect of ANS activation was the tact of the stimuli (Krabs et al., 2015). There was also a statistically significant difference in HRV frequency variables and the flow level during the gait with the first type of stimulation at a slowed and accelerated tempo relative to the natural one, which might be because the participants' body reacted to the change in the tempo of the inflicted stimulation, which was no longer preferred. Changing the sound stimuli from the first to the second stage caused an increase in EDA and HRV rates associated with ANS stimulation. Csikszentmihalyi (1997) proved that flow and ANS stimulation had a frequent effect during the musical experience, which is of importance in the context of the present study.

According to the available literature, the selected variables, both frequency and time-domain determined, are directly reflected in different types of ANS activation:

- SDNN is responsible for the overall HRV estimation and reflects the parasympathetic component of autonomic function,
- pNN50 reflects parasympathetic activity,
- LF consists of a combination of sympathetic and parasympathetic effects and is related to thermoregulation and peripheral vasomotor activity,
- HF relates to the parasympathetic activity of the ANS and modulates this value,
- LF/HR ratio reflects sympathicotonic balance (Krabs et al, 2015; Yilmaz et al., 2018).

The analysis and results obtained showed a statistically significant positive correlation between flow and HRV frequency variables. It is supposed that HRV variables, related to normative ranges, are significantly correlated with the flow state, and indicate the individuals' arousal at the average level. This statement has been confirmed in the literature, as it has also been proven that experiencing flow occurs most often at moderate levels of arousal, not at extremely high levels (Peifer et al., 2014). The study by Peifer et al. (2014) also found a positively correlated relationship between HRV variables (low frequency and high frequency) and experiencing. Other research has proven that the flow experience is a distinct state that can be defined based on declarative information (psychological tests) and physiological data (Keller et al., 2011). The study by Tozman et al. (2015) indicated that LF, HF, and LF/HF ratio variables significantly differed depending on the task's difficulty and correlated with the flow level. De Manzano et al. (2010) confirmed that the increased sensation of flow experience was associated with increased parasympathetic modulation of sympathetic activity and caused an increase in LF/HF values, which could potentially be used as an indicator of flow, particularly during tasks involving music. Additional modalities with high measurement reliability, such as electroencephalography combined with HRV and EDA, are likely to contribute to the higher accuracy of the proposed methods. Obtaining a high degree of convergence of multimodal data would reduce inference error and increase confidence in the results, and combining more than one measurement strategy can achieve higher relevance (validity) in research (Seo et al., 2019).

## Conclusions

The benefit of using data from electrophysiological measurements in conjunction with subjective psychological measures (research triangulation) can deepen insights into the underlying psychological mechanisms that describe responses to external stimuli. There is the possibility of a deeper understanding of the phenomenon under investigation and generating new ways of explaining it. By comparing the analysis of the subjective response of the body to the stimuli, it can be assumed that personalised metrorhythmic stimulation will positively affect the task's expected results in synchronisation of the presented arousal and gait. Therefore, it is important to clarify comprehensive and more accurate models of experienced emotions and objective flow analysis during the task, especially with external stimulation in sound arousal, which could be personalised based on the analysis of musical preferences. A detailed analysis of physiological responses during the performed physical activity will not only provide important information on the level of fatigue of the exercising person but, more importantly, will allow to estimate to what extent the performance of a given activity is related to the individual's positive experience. The present research proves the necessity of monitoring physiological signals during performed exercise as an element of objectification of emotional reactions. It draws attention to crucial variables that allow the modelling of the level of induced flow.

Certainly, the limitations of our study include the analysis of physiological data, which may be disrupted by artefacts caused by movement, and this is not always completely eliminable. Additionally, participants performed the research task for short time, which might not have allowed for full immersion in the experience and could have introduced additional stress. Longer task duration could allow for reaching a deeper level of flow. It is also important to understand whether the performed activity posed a challenge for participants, that is, a situation requiring them to demonstrate skills, knowledge, creativity or other resources, but simultaneously, was within their capabilities. In future, applying the same research protocol to studying professional musicians with well-trained synchronisation between music and movement would be valuable.
